# A patient perspective of the impact of medication side effects on adherence: results of a cross-sectional nationwide survey of patients with schizophrenia

**DOI:** 10.1186/1471-244X-12-20

**Published:** 2012-03-20

**Authors:** Marco DiBonaventura, Susan Gabriel, Leon Dupclay, Shaloo Gupta, Edward Kim

**Affiliations:** 1 Health Sciences Practice, Kantar Health, 11 Madison Avenue, 12th Floor, New York, NY, USA; 2 Health Economics and Outcomes Research, Novartis Pharmaceutical Corporation, East Hanover, NJ, USA

## Abstract

**Background:**

Antipsychotic medications often have a variety of side effects, however, it is not well understood how the presence of specific side effects correlate with adherence in a real-world setting. The aim of the current study was to examine the relationship between these variables among community-dwelling patients with schizophrenia.

**Methods:**

Data were analyzed from a 2007-2008 nationwide survey of adults who self-reported a diagnosis of schizophrenia and were currently using an antipsychotic medication (N = 876). The presence of side effects was defined as those in which the patient reported they were at least "somewhat bothered". Adherence was defined as a score of zero on the Morisky Medication Adherence Scale. To assess the relationship between side effects and adherence, individual logistic regression models were fitted for each side effect controlling for patient characteristics. A single logistic regression model assessed the relationship between side effect clusters and adherence. The relationships between adherence and health resource use were also examined.

**Results:**

A majority of patients reported experiencing at least one side effect due to their medication (86.19%). Only 42.5% reported complete adherence. Most side effects were associated with a significantly reduced likelihood of adherence. When grouped as side effect clusters in a single model, extra pyramidal symptoms (EPS)/agitation (odds ratio (OR) = 0.57, p = 0.0007), sedation/cognition (OR = 0.70, p = 0.033), prolactin/endocrine (OR = 0.69, p = 0.0342), and metabolic side effects (OR = 0.64, p = 0.0079) were all significantly related with lower rates of adherence. Those who reported complete adherence to their medication were significantly less likely to report a hospitalization for a mental health reason (OR = 0.51, p = 0.0006), a hospitalization for a non-mental health reason (OR = 0.43, p = 0.0002), and an emergency room (ER) visit for a mental health reason (OR = 0.60, p = 0.008).

**Conclusions:**

Among patients with schizophrenia, medication side effects are highly prevalent and significantly associated with medication nonadherence. Nonadherence is significantly associated with increased healthcare resource use. Prevention, identification, and effective management of medication-induced side effects are important to maximize adherence and reduce health resource use in schizophrenia.

## Background

Schizophrenia is a chronic, often debilitating psychiatric illness with a lifetime prevalence of approximately 1% of the US population [1,2]. Typically manifesting in late adolescence or early adulthood, schizophrenia can disturb perception, cognition, emotions, and behavior [3]. In addition to pronounced effects on the well-being of patients [4] and their families [5], schizophrenia also exacts an economic burden, estimated at almost $63 billion per year in the US in 2002 [6].

Numerous antipsychotic medications are available with demonstrated efficacy in reducing the acute symptoms of schizophrenia, improving the well-being of patients, and enabling some to live more productive lives [7]. However, adherence and persistence to these medications are important to receive optimal benefits. A review of dropout rates in clinical trials found that 28%-55% of schizophrenia patients drop out of clinical trials before the study is complete; dropout rates were higher with classic antipsychotic medications compared with second generation antipsychotic medications due to side effects [8]. In the Clinical Antipsychotic Trial of Interventional Effectiveness (CATIE) study, 74% of patients discontinued their initial study medication before 18 months [9]. Approximately half of patients with schizophrenia take 70% or less of their medication [10]. Inadequate adherence to antipsychotic medications increases the risk of relapse and associated healthcare utilization and costs [11-13]. A review by Sun et al. (2007) estimated that antipsychotic nonadherence in the US was responsible for between $1.4 and $1.8 billion in rehospitalization costs alone [11].

Studies have suggested that antipsychotic medication side effects are associated with lower levels of adherence [14,15]. Clinicians' ratings of side effects are also associated with treatment discontinuation [16]. Specifically, side effects such as medication-related obesity, distress over weight gain, and cognitive impairment have been associated with increased rates of nonadherence [14,15,17,18]. Although other studies have examined adherence in real-world settings [18], few have studied the relationship between specific side effects and nonadherence. Moreover, there are no studies assessing the relationship between patient-reported side effects and self-reported adherence. This patient perspective is valuable as it provides insight into how the perception of side effects is associated with specific non-adherent behaviors; something that cannot be obtained from objective assessments of adherence. The primary aim of the current study is to assess the relationship between patient-reported antipsychotic side effects and self-reported medication adherence in a community-dwelling sample of patients with schizophrenia. A secondary aim is to assess the relationship between medication adherence and self-reported health resource utilization.

## Methods

### Data source

We analyzed an existing cross-sectional dataset of patients with schizophrenia (N = 1,083). This original survey, conducted between December 2007 and February 2008, was initiated to understand the treatments, attitudes, health behaviors, and health outcomes among patients with schizophrenia. The data generated from this survey has been used in several previous studies, each of which has outlined the methods in some detail [4,19]. Briefly, patients were convenience sampled in one of two ways to participate in a self-administered survey to create the dataset: (1) patients who reported having schizophrenia in an Internet-based consumer panel (Lightspeed Research Ailment Panel) were randomly sent an invitation to participate in a web-based questionnaire via email, and (2) patients were also recruited from grassroots campaigns and newspaper advertising to arrive at a central interview facility to take a paper copy of the survey instrument.

The Lightspeed Research Ailment Panel is an opt-in Internet panel which recruits its members through a variety of online sources (e.g., online support groups, etc.). Patients with schizophrenia who join the panel provide detailed demographic information and agree to participate in a modest number of periodic Internet-based surveys. No sampling frame was used for patients who were recruited outside the Internet panel. Patients who responded to the study advertisements were phone screened for eligibility. Those who reported they were diagnosed with schizophrenia by a healthcare professional, were 18 years of age or older, and were able to read and write English were eligible for this study.

For patients taking the survey online (N = 157), an email address was provided for patients to ask any questions they may have about the survey. Similarly, for patients taking the survey at an interview site (N = 926), a facilitator was present to answer any questions. All patients, regardless of methodology, provided informed consent and were compensated for their participation. Patients who completed the survey online were compensated in the form of points, which can be exchanged for small prizes through Lightspeed Research. Patients who completed the survey offline received a $50 check as compensation. The study protocol and questionnaire were reviewed and approved by Essex IRB (Lebanon, NJ).

### Sample

The study sample was limited to patients who reported that they were currently taking a prescription medication for schizophrenia (N = 876).

### Study measures

#### Patient characteristics

Patient characteristics consisted of demographic data and healthcare characteristics. All demographic data, except age, were assessed as categorical variables and included: gender (female vs. male), ethnicity (non-white vs. white) marital status (married/committed relationship vs. all else), education (some college education or higher vs. no college education/less than college education), employment status (employed full-time or part-time vs. not employed), insurance status (insured vs. uninsured), and poverty (annual household income less than $20,000 vs. income $20,000 or greater). Health characteristics consisted of the total number of comorbid medical conditions, which included type I and type II diabetes, high blood pressure, high cholesterol, heart disease, migraine, liver disease, and HIV.

#### Medication side effects

Medication side effects were self reported. Patients currently taking a prescription medication to treat their schizophrenia were asked, "*In the past month, have you experienced any of the following side effects from your medication?*". Among patients who responded "yes" to any of the listed side effects, they were asked "*how bothered are you by these side effects?*". Responses included "*not at all bothered*", "*not very bothered*", "*somewhat bothered*", "*very bothered*", and "*extremely bothered*". Our pre-specified definition of a side effect included any side effect in which the patient was somewhat, very, or extremely bothered. Side effects were also clustered into five pre-specified categories: "extra pyramidal symptoms (EPS)/agitation" (insomnia, restlessness/feeling jittery, agitation, and tremors); "sedation/cognition" (sedation, difficulty thinking or concentrating, sleepiness, and dizziness); "prolactin/endocrine" (decreased interest in sex, sexual dysfunction, difficult or painful menstrual periods, male breast enlargement or secretions); "metabolic" (weight gain, increase in blood glucose level); and "gastrointestinal (GI)" (nausea/vomiting and constipation). Post-hoc sensitivity analyses used a higher threshold for side effect definition of very or extremely bothered and removed "agitation" from the EPS/agitation cluster due to the potential confounding of agitation and restlessness associated with akathisia.

#### Medication adherence

Adherence to medications was assessed using the four-item Morisky Medication Adherence Scale (MMAS) [20], an instrument which has previously shown to have strong evidence for reliability and validity [20]. The MMAS items include the presence or absence of the following non-adherent behaviors: forgetting to take medication, careless at times about taking medication, stopping medication when feeling better, and stopping medication when feeling worse. For this study we used a threshold of one or more items to identify nonadherence; adherence was classified as reporting "no" for all items. This approach has been reported in previous schizophrenia research [19].

#### Health resource use

Health resource use included self-reported emergency room (ER) visits (for mental and non-mental health-related reasons) and hospitalizations (for mental and non-mental health-related reasons) in the prior six months. Each of these four outcomes was defined dichotomously as yes (1 or more visits) vs. no (0 visits). Prior research has suggested patient-reported measures of resource use show good evidence for validity in comparison with objective measures [21-25].

### Statistical analysis

Descriptive statistics were calculated to describe the patient characteristics, side effect, and medication adherence for the entire sample. Unadjusted comparisons of patient characteristics between adherent and nonadherent groups were conducted using chi-square tests and ANOVA tests for categorical and continuous variables, respectively.

To examine the relationship between side effects and nonadherence, a logistic regression model was fitted for each side effect adjusting for age, gender, ethnicity, education, household income, and number of comorbidities. A single logistic regression model was also fitted using the five pre-specified side effect clusters, controlling the same covariates. To assess the relationship between adherence and health resource use, a logistic regression model was fitted for each of the resource use outcomes, controlling for age, gender, marital status, ethnicity, education, household income, insurance and the number of comorbidities. For all regression models, an a priori threshold for statistical significance was set at p < 0.05.

The individual side effect models were also fitted using the more restrictive definition of side effect presence ("very bothered" or "extremely bothered"). The symptom cluster model was also fitted after removing "agitation" from the EPS/agitation cluster in order to assess the robustness of that cluster.

## Results

The study sample consisted of 876 patients with schizophrenia who reported they were currently taking a medication to treat their condition. The mean age of the sample was 43 years, approximately half of the sample was male, and 61% was white (see Table 1). Over half of the sample was unemployed and 71.7% were taking atypical antipsychotics. The most commonly reported side effects that were at least moderately bothersome included difficulty thinking/concentrating (32.2%), restlessness/feeling jittery (28.2%), insomnia (28.4%), weight gain (25.8%), and sleepiness (25.1%) (see Table 2).

**Table 1:** Sample demographics (N = 876)

	n	%
*Gender*		
Male	432	49.3
Female	444	50.7
*Ethnicity*		
White	536	61.2
Non-white	340	38.8
*Education*		
Less than college	421	48.1
Some college or higher	455	51.9
*Household income*		
Less than $20,000	506	57.8
$20,000 or more	370	42.2
*Marital status*		
Single	665	75.9
Married/living with partner	211	24.1
*Insurance*		
Managed care (HMO, PPO)	241	27.5
Medicare	364	41.6
Veterans Affairs Medical Center	41	4.7
State Medicaid (MediCal for CA residents)	350	40.0
No health insurance	61	7.0
Don't know	16	1.8
*Employment*		
Employed full time	129	14.7
Employed part time	163	18.6
Student	29	3.3
Retired	70	8.0
Not employed	484	55.3
*Atypical Medication*		
On a atypical medication	628	71.7
	**Mean**	**SD**
Age	43.03	11.80
Number of comorbidities	1.27	1.30

**Table 2:** Medication side effects reported by current medication users (N = 876)

	Side effect present and bothersome	
	**n**	**%**

Difficulty thinking/concentrating	349	32.2
Restlessness/feeling jittery	305	28.2
Insomnia	307	28.4
Sleepiness	272	25.1
Weight gain	279	25.8
Decreased interest in sex	223	20.6
Agitation	240	22.2
Sedation	173	16.0
Dizziness	193	17.8
Constipation	175	16.2
Tremors	142	13.1
Sexual dysfunction	136	12.6
Nausea/vomiting	110	10.2
Difficult/painful menstrual periods	51	4.7
Male breast enlargement or secretions	15	1.4
Increased in blood glucose level	9	0.8

**Figure 1:**
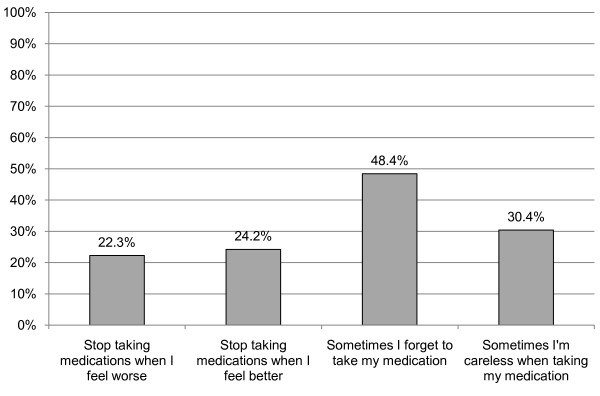
**Prevalence of non-adherent behaviors from the Morisky Medication Adherence Scale**.

The frequency of nonadherent behaviors (individual items of the MMAS) are presented in Figure 1. Nearly half of patients reported that they sometimes forget to take their medication (48.4%). Only 42.5% of patients responded "no" to all four nonadherent behaviors in the MMAS. Table 3 summarizes bivariate comparisons between characteristics of adherent and nonadherent patients. Fewer adherent patients were married or had any college education. There were no other significant differences in patient characteristics between groups.

**Table 3:** Sociodemographic and patient characteristic differences between those adherent and non-adherent with their medication for their schizophrenia

	Non-adherent (n = 504)		Adherent (n = 372)		
	**n**	**%**	**n**	**%**	**p**

Female	260	51.6	184	49.5	0.5344
Married	138	27.4	73	19.6	0.0069
Non-white	192	38.1	148	39.8	0.6127
Some college or higher	283	56.2	172	46.2	0.0037
Household income $20,000 or more	226	44.8	144	38.7	0.0685
Insured	455	90.3	344	92.5	0.2487
Employed	156	31.0	136	36.6	0.0838
	**Mean**	**SD**	**Mean**	**SD**	**p**
Age	42.40	12.02	43.89	11.46	0.0651
Number of comorbidities	1.34	1.29	1.18	1.31	0.0861

**Figure 2:**
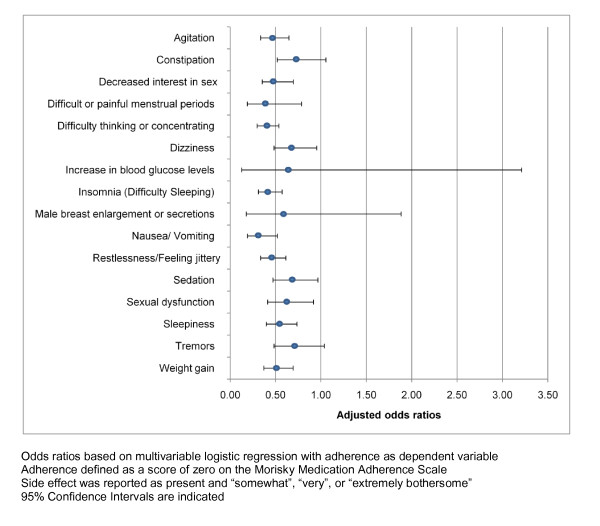
**Adjusted odds ratios for the impact of each side effect on complete adherence**. Odds ratios based on multivariable logistic regression with adherence as dependent variable. Adherence defined as a score of zero on the Morisky Medication Adherence Scale. Side effect was reported as present and "somewhat", "very", or "extremely bothersome". 95% Confidence Intervals are indicated.

The results of the logistic regression models for each side effect are presented in Figure 2. Most of the side effects assessed were significantly associated with a decreased likelihood of medication adherence. Sensitivity analyses using the more restrictive definition of side effect presence did not change overall model results with respect to significance or magnitude (see Additional file 1: Figure S1).

Table 4 summarizes results of the logistic regression model using five side effect clusters. Younger age, unemployment, and higher education were all associated with a lower likelihood of adherence. Similarly, all of the side effect clusters except GI were associated with a lower likelihood of adherence. The EPS/agitation cluster had the strongest effect, with a 43% reduction in odds of being adherent, followed by metabolic side effects with a 36% reduction. Results of the sensitivity analysis removing agitation from the EPS/agitation cluster did not affect the significance or magnitude of the effect (see Additional file 2: Table S1).

**Table 4:** The relationship between side effect clusters and complete medication adherence

	OR	95% LCL	95% UCL	p
Female	1.08	0.80	1.45	0.6209
Married	0.73	0.51	1.06	0.0942
Age	1.02	1.00	1.03	0.0283
Non-White	0.99	0.73	1.33	0.9244
Some college or higher	0.72	0.53	0.97	0.0324
Insured	1.33	0.79	2.24	0.2821
Employed	1.45	1.03	2.03	0.0313
Household income $20,000 or more	0.83	0.60	1.15	0.264
Number of comorbidities	0.96	0.86	1.09	0.549
Agitation/EPS	0.57	0.41	0.78	0.0007
Sedation/Cognition	0.70	0.50	0.97	0.0331
Prolactin/Endrocrine	0.69	0.49	0.97	0.0342
Metabolic	0.64	0.46	0.89	0.0079
GI	0.79	0.55	1.11	0.1729

Patients reporting complete medication adherence were significantly less likely to report a hospitalization for a mental health reason (OR = 0.51; 95%CI: 0.35-0.75, p = .0006), a hospitalization for a non-mental health reason (OR = 0.43; 95%CI: 0.28-0.67, p = .0002), or an ER visit for a mental health reason (OR = 0.60; 95%CI: 0.41-0.87, p = .008).

## Discussion

In this cross-sectional survey of patients in the US with schizophrenia, nearly 80% of patients reported at least one side effect that was at least somewhat bothersome to them, and less than half of patients reported complete adherence to their medications. There were few differences in the characteristics of adherent and nonadherent patients. However, we observed consistently strong relationships between medication side effects and nonadherence. Our finding that cognitive-related side effects and weight gain were associated with nonadherence is consistent with prior research [17,18].

EPS/agitation-related side effects were the most strongly associated with nonadherence, and were commonly reported. This is a striking finding because atypical antipsychotics are generally thought to have lower risk for EPS compared to typical antipsychotics [26,27]. Though, it should be noted, not all patients in this study were on an atypical medication the vast majority were. Both EPS and endocrine side effects of antipsychotic medications are mediated by dopamine receptor 2 (D2) blockade [28]. The mechanism behind metabolic side effects to antipsychotics is less clear, but may be related to histaminergic blockade, which is also implicated in sedative side effects [28,29].

Aside from side effects, a few other variables were significantly associated with adherence. Specifically, patients who were older, less educated, and employed were more likely to be adherent. Prior evidence has suggested that longer illness duration is associated with greater adherence [30]; therefore, age may be serving as a proxy for years diagnosed. Employment may be serving as a proxy for level of functioning (i.e., patients with improved functioning are more likely to be in the labor force), as poor disease insight has been shown to be associated with poorer adherence [30]. Interestingly, few studies have uncovered a relationship between education and adherence. Because of the lack of support in the literature, it is possible education also serves as a proxy for another unmeasured variable (e.g., negative attitudes toward medications), though additional research is warranted.

Consistent with previous studies [11], nonadherence is a significant risk factor for hospital and emergency room use. In our study, we found that both mental health and non-mental health hospital use was increased in nonadherent patients. Antipsychotic medications demonstrate high variability in their risk of inducing various side effects, and this may be mediated by differential affinities for D2, 5HT2A, and other receptors [28,29]. Clinicians may be faced with the challenge of choosing between medications with a lower risk for EPS yet higher risk for metabolic complications, and vice versa, but we find that both side effects are associated with significant nonadherence that may lead to both psychiatric and non-psychiatric hospitalizations. Therefore, preventing, identifying, and minimizing the frequency and severity of medication-related side effects may lead to greater adherence and fewer hospitalizations.

### Limitations

This study has several limitations. Because all data were self-reported, diagnoses, treatments, adherence levels, and healthcare resource utilization were not confirmed by clinicians, patient records, or administrative claims data. Patients may have either underreported or overreported their experience with side effects (perhaps attributing a medication side effect to a symptom of a comorbid condition or vice versa) and their level of adherence. However, this patient perspective can still be valuable. Regardless of the true reason for the experienced "side effect", a patient's perception of the reason for the side effect may be more important in predicting adherence. Even if the side effect is not due to their schizophrenia medication, attributing it as such can result in greater non-adherence.

The cross-sectional design prevents robust ascertainment of causality. Although the hypothesis was that the presence of side effects leads to greater non-adherence it is possible that non-adherence (particularly sudden medication suspension followed by a full dose) can increase the presence of side effects. It is also possible that increasing adherence may lead to more side effects.

It is possible that unobserved confounding may have influenced the observed results. For example, severity of schizophrenia, polypharmacy, complexity of medication regimen, medication costs, among other variables, are likely associated with non-adherence but were not included in the current study. Similarly, limited information was available with respect to the number of non-antipsychotic medications. The greater the number of medications, the more difficult it may be for patients to determine which side effects are due to which treatments. Finally, the use of a convenience sample may have resulted in a sample that does not generalize to the community-dwelling population of patients with schizophrenia, as all patients were willing and functionally able to participate in survey research. The usage of both online and offline sample sources was implemented to ensure variability in patient types but these patient types may not be representative of the larger population.

## Conclusions

Side effects of antipsychotic medications are highly prevalent and significantly associated with lower adherence, which is associated with increased healthcare resource use. Prevention of, monitoring for, and minimizing medication side effects may lead to better adherence and improved outcomes.

## Abbreviations

CATIE: Clinical antipsychotic trial of interventional effectiveness; IRB: Institutional review board; EPS: Extra pyramidal symptoms; GI: Gastrointestinal; MMAS: Morisky medication adherence scale; ER: Emergency room; ANOVA: Analysis of variance; D2: Dopamine receptor 2.

## Competing interests

The nationwide survey reported here was completed by Kantar Health. Novartis Pharmaceutical Corporation purchased access to the data of the survey and funded the analysis and preparation of this manuscript. Ms. Gupta and Dr. DiBonaventura are current employees of Kantar Health; Ms. Gabriel and Drs. Dupclay and Kim are current employees of Novartis Pharmaceutical Corporation.

## Authors' contributions

All authors (MD, SGa, LD, SGu, and EK) helped contribute to the conception and design of the current study. MD and SGu performed the statistical analyses. All authors helped contribute to the interpretation of the results. MD and SGu drafted the manuscript. All authors provided critical revisions to the various manuscript drafts. All authors read and approved the final manuscript

## Pre-publication history

The pre-publication history for this paper can be accessed here:

http://www.biomedcentral.com/1471-244X/12/20/prepub

## Supplementary Material

Additional file 1 **Adjusted odds ratios for the impact of each side effect on complete adherence with more restrictive definition of side effect presence**. Click here for file

Additional file 2 **The relationship between side effect clusters and complete medication adherence, removing agitation from the agitation/EPS cluster**. Click here for file

## References

[B1] Kendler KS Gallagher TJ Abelson JM Kessler RC Lifetime prevalence, demographic risk factors, and diagnostic validity of nonaffective psychosis as assessed in a US community sample. The National Comorbidity Survey Arch Gen Psychiatry 1996 53 1022 1031 10.1001/archpsyc.1996.01830110060007 8911225

[B2] Goldner EM Hsu L Waraich P Somers JM Prevalence and incidence studies of schizophrenic disorders: a systematic review of the literature Can J Psychiatry 2002 47 833 843 12500753 10.1177/070674370204700904

[B3] Messias EL Chen CY Eaton WW Epidemiology of schizophrenia: review of findings and myths Psychiatr Clin North Am 2007 30 323 338 10.1016/j.psc.2007.04.007 17720026 PMC2727721

[B4] DiBonaventura MD Panish J Kenworthy D Wagner J-S Dirani R The association of well-being, productivity and resource use among community-dwelling patients with schizophrenia using atypical antipsychotics J Pharmaceut Health Serv Res 2010 1 181 187 10.1111/j.1759-8893.2010.00033.x

[B5] Caqueo-Urizar A Gutierrez-Maldonado J Miranda-Castillo C Quality of life in caregivers of patients with schizophrenia: a literature review Health Qual Life Outcomes 2009 7 84 10.1186/1477-7525-7-84 19747384 PMC2749816

[B6] Wu EQ Birnbaum HG Shi L Ball DE Kessler RC Moulis M Aggarwal J The economic burden of schizophrenia in the United States in 2002 J Clin Psychiatry 2005 66 1122 1129 10.4088/JCP.v66n0906 16187769

[B7] Liberman RP Kopelowicz A Ventura J Gutkind D Operation criterial and factors related to recovery from schizophrenia Int Rev Psychiatry 2002 14 256 272 10.1080/0954026021000016905

[B8] Kemmler G Hummer M Widschwendter C Fleischhacker WW Dropout rates in placebo-controlled and active-control clinical trials of antipsychotic drugs: a meta-analysis Arch Gen Psychiatry 2005 62 1305 1312 10.1001/archpsyc.62.12.1305 16330718

[B9] Lieberman JA Stroup TS McEvoy JP Swartz MS Rosenheck RA Perkins DO Keefe RS Davis SM Davis CE Lebowitz BD Effectiveness of antipsychotic drugs in patients with chronic schizophrenia N Engl J Med 2005 353 1209 1223 10.1056/NEJMoa051688 16172203

[B10] Goff DC Hill M Freudenreich O Strategies for improving treatment adherence in schizophrenia and schizoaffective disorder J Clin Psychiatry 2010 71 Suppl 2 20 26 10.4088/JCP.9096su1cc.04 21190649

[B11] Sun SX Liu GG Christensen DB Fu AZ Review and analysis of hospitalization costs associated with antipsychotic nonadherence in the treatment of schizophrenia in the United States Curr Med Res Opin 2007 23 2305 2312 10.1185/030079907X226050 17697454

[B12] Nicholl D Akhras KS Diels J Schadrack J Burden of schizophrenia in recently diagnosed patients: healthcare utilisation and cost perspective Curr Med Res Opin 2010 26 943 955 10.1185/03007991003658956 20163295

[B13] Knapp M Locklear J Jarbrink K Impact of psychotic relapse definitions in assessing drug efficacy and costs: comparison of quetiapine XR, olanzapine and paliperidone ER Curr Med Res Opin 2009 25 1593 1603 10.1185/03007990903010623 19469696

[B14] Barbui C Kikkert M Mazzi MA Becker T Bindman J Schene A Nose M Helm H Thornicroft G Tansella M Comparison of patient and clinician perspectives in the assessment of antipsychotic medication adherence Psychopathology 2009 42 311 317 10.1159/000232973 19672133

[B15] Burton SC Strategies for improving adherence to second-generation antipsychotics in patients with schizophrenia by increasing ease of use J Psychiatr Pract 2005 11 369 378 10.1097/00131746-200511000-00003 16304505

[B16] Fenton WS Blyler CR Heinssen RK Determinants of medication compliance in schizophrenia: empirical and clinical findings Schizophr Bull 1997 23 637 651 10.1093/schbul/23.4.637 9366000

[B17] Weiden PJ Mackell JA McDonnell DD Obesity as a risk factor for antipsychotic noncompliance Schizophr Res 2004 66 51 57 10.1016/S0920-9964(02)00498-X 14693352

[B18] Ascher-Svanum H Zhu B Faries D Lacro JP Dolder CR A prospective study of risk factors for nonadherence with antipsychotic medication in the treatment of schizophrenia J Clin Psychiatry 2006 67 1114 1123 10.4088/JCP.v67n0715 16889456

[B19] Kim E Gupta S Bolge S Chen CC Whitehead R Bates JA Adherence and outcomes associated with copayment burden in schizophrenia: a cross-sectional survey J Med Econ 2010 13 185 192 10.3111/13696991003723023 20235753

[B20] Morisky DE Green LW Levine DM Concurrent and predictive validity of a self-reported measure of medication adherence Med Care 1986 24 67 74 10.1097/00005650-198601000-00007 3945130

[B21] Goossens ME Rutten-van Molken MP Vlaeyen JW van der Linden SM The cost diary: a method to measure direct and indirect costs in cost-effectiveness research J Clin Epidemiol 2000 53 688 695 10.1016/S0895-4356(99)00177-8 10941945

[B22] Petrou S Murray L Cooper P Davidson LL The accuracy of self-reported healthcare resource utilization in health economic studies Int J Technol Assess Health Care 2002 18 705 710 12391960 10.1017/s026646230200051x

[B23] Pinto D Robertson MC Hansen P Abbott JH Good agreement between questionnaire and administrative databases for health care use and costs in patients with osteoarthritis BMC Med Res Methodol 2011 11 45 10.1186/1471-2288-11-45 21489280 PMC3095571

[B24] Raina P Torrance-Rynard V Wong M Woodward C Agreement between self-reported and routinely collected health-care utilization data among seniors Health Serv Res 2002 37 751 774 10.1111/1475-6773.00047 12132604 PMC1434660

[B25] Ritter PL Stewart AL Kaymaz H Sobel DS Block DA Lorig KR Self-reports of health care utilization compared to provider records J Clin Epidemiol 2001 54 136 141 10.1016/S0895-4356(00)00261-4 11166528

[B26] Haro JM Salvador-Carulla L The SOHO (Schizophrenia Outpatient Health Outcome) study: implications for the treatment of schizophrenia CNS Drugs 2006 20 293 301 10.2165/00023210-200620040-00003 16599647

[B27] Luft B Taylor D A review of atypical antipsychotic drugs versus conventional medication in schizophrenia Expert Opin Pharmacother 2006 7 1739 1748 10.1517/14656566.7.13.1739 16925501

[B28] Nasrallah HA Atypical antipsychotic-induced metabolic side effects: insights from receptor-binding profiles Mol Psychiatry 2008 13 27 35 10.1038/sj.mp.4002066 17848919

[B29] Stahl SM Describing an atypical antipsychotic: receptor binding and its role in pathophysiology Prim Care Companion J Clin Psychiatr 2003 5 Suppl 3 9 13

[B30] Lacro J Dunn L Dolder C Leckband SG Jeste DV Prevalence of and risk factors for medication nonadherence in patients with schizophrenia: a comprehensive review of recent literature J Clin Psychiatry 2002 63 892 908 10.4088/JCP.v63n1007 12416599

