# Supplementary material for: A patient perspective of the impact of medication side effects on adherence: results of a cross-sectional nationwide survey of patients with schizophrenia
Source: BMC Psychiatry. 2012 Mar 20;12:20. doi: 10.1186/1471-244X-12-20 (PMC3342101; doi:10.1186/1471-244X-12-20)
Supplement: Additional file 1 — Adjusted odds ratios for the impact of each side effect on complete adherence with more restrictive definition of side effect presence. [file 1471-244X-12-20-S1.PDF]

Additional file 1: Adjusted odds ratios for the impact of each side effect on complete adherence with more restrictive definition of side effect presence.

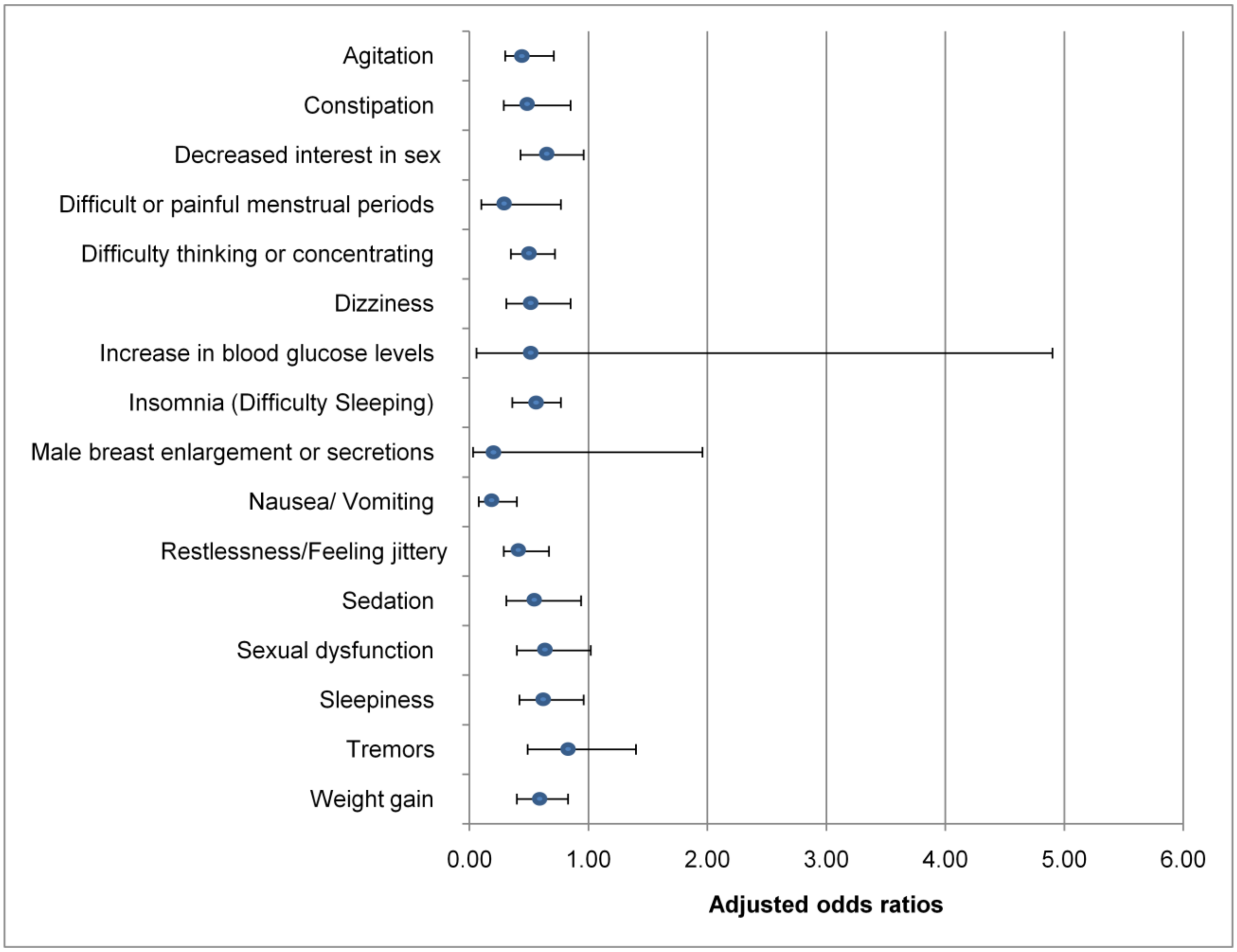

Odds ratios based on multivariable logistic regression with adherence as dependent variable
Adherence defined as a score of zero on the Morisky Medication Adherence Scale
Side effect was reported as present and “somewhat”, “very”, or “extremely bothersome”
95% Confidence Intervals are indicated
