# Supplementary material for: A patient perspective of the impact of medication side effects on adherence: results of a cross-sectional nationwide survey of patients with schizophrenia
Source: BMC Psychiatry. 2012 Mar 20;12:20. doi: 10.1186/1471-244X-12-20 (PMC3342101; doi:10.1186/1471-244X-12-20)
Supplement: Additional file 2 — The relationship between side effect clusters and complete medication adherence, removing agitation from the agitation/EPS cluster. [file 1471-244X-12-20-S2.PDF]

Additional file 2: The relationship between side effect clusters and medication adherence, removing agitation from the agitation/EPS cluster.

	OR (95%CI)	p
Female	1.08 (0.80,1.45)	0.6248
Married	0.74 (0.51,1.06)	0.101
Age	1.02 (1.00,1.03)	0.0277
Non-White	0.99 (0.74,1.33)	0.9496
Some college or higher	0.72 (0.53,0.97)	0.0302
Insured	1.34 (0.79,2.25)	0.275
Employed	1.45 (1.04,2.03)	0.0299
Household income $20,000 or more	0.82 (0.59,1.14)	0.238
Number of comorbidities	0.96 (0.86,1.08)	0.5283
EPS	0.62 (0.44,0.85)	0.0035
Sedation/Cognition	0.67 (0.48,0.93)	0.0179
Prolactin/Endocrine	0.68 (0.48,0.95)	0.0242
Metabolic	0.64 (0.46,0.89)	0.0073
GI	0.79 (0.55,1.11)	0.1726

OR= Odds ratio based on multivariable logistic regression with adherence as dependent variable
Adherence defined as a score of zero on the Morisky Medication Adherence Scale
95% CI = 95% Confidence Interval
EPS=Extrapyramidal symptoms
GI = Gastrointestinal
